# Bridging the gap between self‐assessment and faculty assessment of clinical performance in restorative dentistry: A prospective pilot study

**DOI:** 10.1002/cre2.567

**Published:** 2022-04-06

**Authors:** Ghaith Alfakhry, Khattab Mustafa, M. Abdulhadi Alagha, Hussam Milly, Mayssoon Dashash, Issam Jamous

**Affiliations:** ^1^ Faculty of Dentistry Damascus University Damascus Syria; ^2^ Program of Medical Education Syrian Virtual University Damascus Syria; ^3^ Department of Restorative Dentistry, Faculty of Dentistry Damascus University Damascus Syria; ^4^ Department of Orthopedics Institute of Global Health Innovation, Imperial College London London UK; ^5^ Department of Pedodontics, Faculty of Dentistry Damascus University Damascus Syria; ^6^ Department of Fixed Prosthodontics, Faculty of Dentistry Damascus University Damascus Syria

**Keywords:** dental education, restorative dentistry, self‐assessment, self‐regulated learning

## Abstract

**Purpose/Objectives:**

The current study was undertaken to investigate whether self‐assessment of clinical skills of undergraduate dental students could be bridged with faculty assessment by deliberate training over an extended period. A secondary aim was to explore students' perception of self‐assessment and its effect on their learning and motivation.

**Material and Methods:**

A prospective pilot study was conducted at the Department of Restorative Dentistry at Damascus University. Sixteen students participated in the study, ranging in age between 22 and 23 years. A modified Direct Observation of Procedural Skills form with a grading rubric was used to register and guide students' self‐assessment; both were pretested on four students before the study. In total, four clinical encounters were completed by each student. Students were trained on how to conduct proper self‐assessment before and after each clinical encounter. A postcourse questionnaire was used to investigate students' perception of self‐assessment.

**Results:**

Bias in self‐assessment decreased consistently after each encounter, and the difference in bias between the first (bias = 0.77) and the last encounter (bias = 0.21) was significant with a medium effect size (*p* = .022, *d* = 0.64). The percentage of disconfirming performance dimensions decreased from 39.7% to 26.9%. Students' ability to exactly pinpoint strengths improved consistently and significantly. However, their ability to pinpoint areas of improvement was volatile and showed no significant difference. Bland–Altman graph plots showed higher levels of agreement between self‐assessment and faculty assessment. Moreover, students' perception of self‐assessment was very positive overall.

**Conclusions:**

These findings suggest the possibility that the gap between self‐assessment and faculty assessment could be bridged through deliberate training. Future longitudinal research using a larger sample size is still required to further explore whether self‐assessment can be actively bridged with faculty assessment by deliberate training.

## 1 INTRODUCTION

Self‐assessment is a common metacognitive feature that requires a proactive learner who can demonstrate self‐awareness and collect needed data to reliably assess his or her own performance on a specific task (Cleary et al., 2013). Reliable self‐assessment can facilitate self‐regulated learning (SRL) by helping learners determine learning goals and areas of improvement. A more comprehensive conceptualization of self‐assessment was provided by Epstein et al. (2008): “Self‐assessment is a process of interpreting data about our own performance and comparing it to an explicit or implicit standard.” This definition places emphasis on having high‐quality external standards for conducting an accurate objective self‐assessment in contrast to performing a self‐assessment based on implicit personal standards. A Commission on Dental Accreditation (2013) in the United States had placed emphasis on educational programs that focus on fostering self‐assessment and SRL skills.

A number of studies reported the use of self‐assessment in the undergraduate and postgraduate dental curricula in many different preclinical subjects such as dental anatomy (Abdalla et al., 2021; Nance et al., 2009) as well as clinical subjects such as fixed and removable prosthodontics (Chambers & LaBarre, 2014; Cho et al., 2010; Saadé et al., 2021), geriatric dentistry (Patel et al., 2020), and operative dentistry (Mays & Levine, 2014); few studies have focused on students' self‐assessment of communication skills (Cuevas‐Nunez et al., 2022; Lanning et al., 2011). Most of the studies in the literature showed that dental students lack the ability to perform an accurate self‐assessment (Evans et al., 2007; Mays & Levine, 2014), and this finding was also reproduced in medical education research (Eva & Regehr, 2011; Wieck et al., 2018). Several attempts have been made to improve students' self‐assessment accuracy; however, no significant improvement was detected (Satheesh et al., 2015). It has been assumed that self‐assessment may be a stable attribute that matures during childhood and stops progressing by the time students reach medical school (T. Fitzgerald, 1997). A longitudinal study of self‐assessment accuracy in medical education supports the notion that self‐assessment accuracy is relatively stable (J. T. Fitzgerald et al., 2003), and there is also some evidence of this in dental education (Curtis et al., 2008; Satheesh et al., 2015). Nevertheless, there have been suggestions that these results reflect the little to no practice that students receive in self‐assessment during their education (J. T. Fitzgerald et al., 2003).

According to a systematic review of the use of self‐assessment in dental education (Mays & Branch‐Mays, 2016), many studies did not use a structured assessment form or a criteria sheet, which shows students what the desired outcome should look like. Moreover, studies were limited to a single encounter and did not provide much information regarding any structured training of students on how to self‐assess and also did not provide information on students' attitudes and perception of self‐assessment activities.

This study investigated whether self‐assessment of clinical skills of undergraduate dental students could be bridged with experienced faculty assessment through deliberate training on this skill over an extended period. The null hypothesis was that there would be no significant difference in self‐assessment indicators in comparison with faculty assessment between the start of the training and at the end. A secondary aim of this study is to qualitatively investigate students' perception of self‐assessment and its impact on learning and motivation.

## 2 METHODS

This study was granted ethical approval on December 20, 2020 by the Faculty of Dentistry at Damascus University (number: 85817). Participation in this study was voluntary and confidential, and participants were able to withdraw at any stage of the study.

### 2.1 Study design

This is a prospective pilot study that was conducted at the Department of Restorative Dentistry at the Faculty of Dentistry, Damascus University, in Syria. The study was undertaken during the entire second term of the academic year 2020/2021, starting in late March 2021 and ending in late June 2021.

### 2.2 Participants and settings

Students were invited to participate via an online questionnaire in which students were screened and provided their consent. Sixteen students participated, 11 (68.7%) of whom were females; this number was reasonable on the basis of the available human and financial resources. Participants' age range was between 22 and 23 years. The participants in the study comprised a convenience sample size. Six faculty in total were assigned. Each student had to complete four self‐assessment encounters under the supervision of qualified dentists, who were third‐year postgraduate students in Restorative Dentistry. Students' clinical performance skills were evaluated before the study by their previous clinical faculty as novice in restorative dentistry. Students' scores in the piloted assessment method did not affect their official grades in the module, and this was made clear to the students before participation.

### 2.3 Data collection

#### 2.3.1 Assessment tool development

A validated Direct Observation of Procedural Skills (DOPS) form design was used as a template (Tricio et al., 2015), and another assessment tool in restorative dentistry helped shape the final assessment form and the grading rubric (Dilbone et al., 2016). The general additions and changes to the form can be found in the Supporting Information.

**Figure 1:**
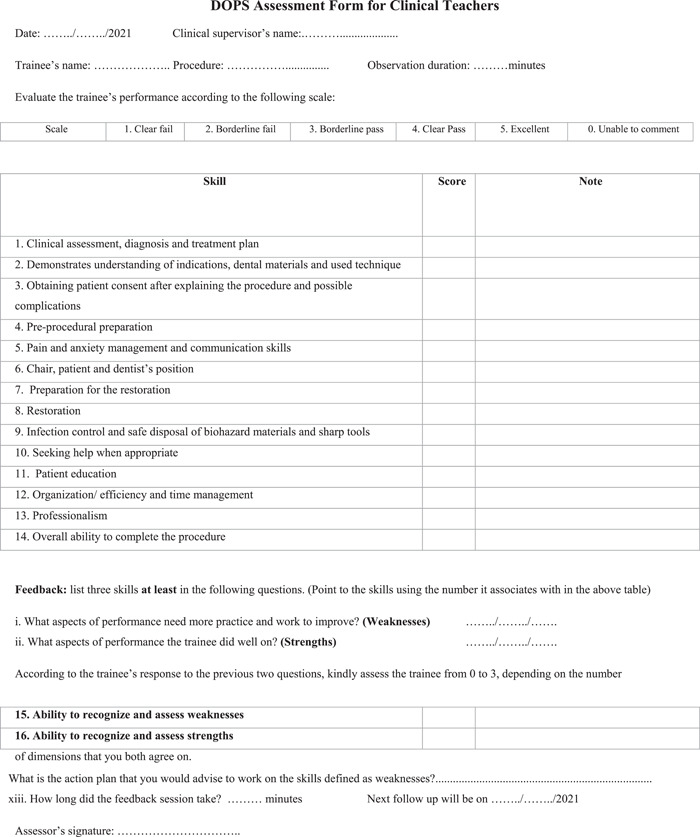
DOPS assessment form for clinical teachers. Students' assessment form was the same as the clinical teachers' form, except for Items 15 and 16. DOPS, Direct Observation of Procedural Skills

Certain essential modifications were made to the feedback section (strengths and areas of improvement identification); instead of making the questions open‐ended, they were transformed into closed‐ended as seen in the form (Figure 1). An extra open‐ended question was added so that *an action plan* is designed to address areas of improvement (iii, Figure 1). In the original template form, students' ability to identify areas of improvement and strengths was assessed in a single dimension (item), whereas in the current study, they were assessed exclusively by faculty in two separate dimensions. The argued rationale behind this is that trainees might be less able to identify areas of improvement than strengths. Since the difficulty level of cases was standardized between students to be medium, the case complexity item was omitted. The range of procedures that the students performed were amalgam or composite restoration Class I, II, III, IV, or V. The procedures, case difficulty, materials, and time are all variables that were standardized between students and across encounters.

A note section was left next to each assessment domain (item) in order to allow students and faculty to write down the specific observations that guaranteed a certain given grade. To make the assessment more reliable and valid, an analytical grading rubric was specifically designed for the used DOPS form (Jonsson & Svingby, 2007). This also helped make the assessment criteria explicit to both students and faculty, thus enabling feedback and self‐assessment (Almohaimede, 2021; Jonsson & Svingby, 2007).

The final global form (Figure 1) included three clinical performance domains (knowledge, skills, attitudes); under each, there were a number of dimensions (items). Items 1, 2, and 10 were under the knowledge domain, Items 6, 7, and 8 were under the skills domain, and the rest were under the attitude domain. The assessment form, grading rubric, and the assessment protocol were pretested on four students to check adherence to general guidelines and after that, some assessment items were rephrased to improve clarity in accordance with faculty and students' feedback.

#### 2.3.2 Assessor calibration and students' self‐assessment training

**Figure 2:**
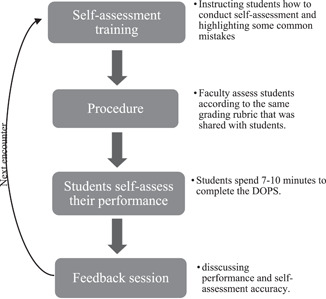
A flowchart showing the self‐assessment training process in each clinical encounter that students had in the study

Faculty were trained to assess using the designed DOPS and grading rubric to ensure maximum interrater reliability. A precourse session was given to both faculty and students separately on how to conduct DOPS using the grading rubric. The assessment form and the grading rubric were provided with clear instructions in electronic format to students before the clinical course, and faculty had a hard copy with them all the time. Figure 2 illustrates the sequence of the self‐assessment training process in each DOPS encounter.

Before each assessment encounter, a 5‐min discussion session was conducted to highlight notes and strategies in conducting an objective self‐assessment; the common mistakes were highlighted and students were instructed to adhere to the grading criteria as much as possible. Students had to complete the DOPS form after they finished the procedure (retrospectively), whereas faculty were instructed to assess students during the procedure. Upon handing in the forms, a short 5‐min feedback session took place. Faculty prompted students to elaborate on the reasoning process behind their self‐assessment by asking questions such as “Why did you give yourself an x‐grade on this skill?” These questions addressed if there were discrepancies in grading and ensured that students are mindfully reflecting on their performance. Faculty did not provide instructions or interfere during students' clinical work unless patients' safety could be compromised.

The study used a validated DOPS form that was designed specifically for dental students (Tricio et al., 2015). All modifications, which were mainly minor, were approved by a panel of experts in restorative dentistry and dental education and were pretested before administration. All provided documents were validated and written in English and then translated into Arabic by qualified translators, who also conducted backward translation to ensure translation accuracy. The final Arabic forms were presented again to a panel of experts in restorative dentistry, who approved them.

#### 2.3.3 Self‐assessment measurement

The overall score of self‐assessment and faculty assessment was calculated as the mean score of the three domains (knowledge, skills, attitude), and each domain mean score was measured by calculating the mean score of their respective dimensions. The mean difference between the self‐assessment and the faculty assessment was used as the *Bias* indicator. Items 15 and 16 were used as indicators of students' ability to identify areas of improvement and strengths, and they were assessed based on the number of dimensions that students and faculty agreed on; the minimum score is 0 (total disagreement) and the maximum score is 3 (total agreement). For instance, if the student response to “i” was 1/5/10 and the faculty response was 1/6/10, the student's ability to identify areas of improvement will be assessed as two out of three.

Another measurement method of self‐assessment was the disconfirming dimensions percentage (DDP), which indicates the percentage of dimensions, where the students' self‐assessment was disconfirming to the faculty assessment. Self‐assessment was considered disconfirming at a certain dimension when it was (pass: 3 or 4) and the faculty assessment was (fail: 2 or 1), or the self‐assessment score was (excellent: 5) and faculty assessment was ≤3 (borderline pass) or vice versa. The DDP was calculated to measure whether certain or different dimensions accounted for the variance in the *Bias* score.

#### 2.3.4 Investigating students' perception of self‐assessment

Students assessed the utility of feedback after each DOPS encounter on a 5‐point scale, and an online postcourse questionnaire was used to assess students' attitudes, perceived educational impact, and the challenges of the assessment method; both closed‐ and open‐ended questions were used. The responses to closed‐ended were on a 5‐point Likert scale. Students' responses were first written in Arabic and then translated into English by qualified translators.

### 2.4 Data analysis

A paired *t* test was used to compare the difference in means (*Bias*) between self‐assessment and faculty assessment. The difference between self‐assessment measures in the first encounter and the last (fourth) encounter was analyzed using the paired *t* test, and Cohen's *d* was used as a measure of effect size (Cohen, 2013). Bland–Altman plots were used to analyze the agreement between self‐assessment and the faculty assessment in each encounter and 95% limits of agreement were calculated accordingly; the correlation coefficient was not used as it describes the linear relationship between two variables (Udovičić et al., 2007), but not their agreement as the Bland–Altman plot does (Bland & Altman, 1999). The Shapiro–Wilk test was used to check the normal distribution of data before conducting a paired *t* test and constructing Bland–Altman plots. As for the measures of students' ability to identify areas of improvement and strengths, the data were ordinal and were treated as such, so the Wilcoxon signed‐rank test was used to analyze the difference between encounters. Thematic analysis, which was recommended by Field and Morse (1985), was used to analyze students' responses on the open‐ended questions in the postcourse questionnaire.

Statistical analysis was performed using SPSS 26 (IBM SPSS Statistics for Windows, 2019), and Microsoft Excel (2016) was used to process data and calculate Cohen's *d*. The online postcourse questionnaire survey was conducted on Google Forms.(Google). As for qualitative data coding and analysis, MAXQDA 2020 was used (MAXQDA 2020, 2019).

## 3 RESULTS

### 3.1 The difference between self‐assessment and faculty assessment

Table 1 shows descriptive statistics of bias and DDP along with the mean score of self‐assessment and faculty assessment in each of the four encounters. The positive bias value indicated that self‐assessment was higher on average than the faculty assessment. Overall, indicators showed a decrease in the gap between self‐assessment and faculty assessment. The bias score was statistically significant in the first encounter (Bias = 0.77, *p* < .001). However, in the fourth encounter, the bias decreased by about 70% in comparison with the first, with no statistically significant difference between the self‐assessment and the faculty assessment. The bias was also significant in the third encounter; however, its confidence interval was narrower than the second encounter. The difference in bias between the first and the last encounter was significant with a medium effect size (*p* = .022, *d* = 0.64) (Table 2). The percentage of disconfirming dimensions decreased from 39.7% in the first encounter to 26.9% in the fourth encounter.

**Table 1:** Means of self‐assessment measures (Bias, DDP) in each of the four encounters along with self‐assessment and faculty assessment

	*n*	1st encounter (95% CI)	2nd encounter (95% CI)	3rd encounter (95% CI)	4th encounter (95% CI)
Bias	16	0.77*** (1.1−0.4)	0.35 (0.8 to −0.1)	0.36* (0.7−0.002)	0.21 (0.4 to −0.01)
Disconfirming dimensions percentage	16	39.7% (29.9−49.6)	31.6% (20.4−42.7)	33.3% (27.5−39.0)	26.9% (19.2−34.5)
Faculty assessment	16	2.9 (2.6−3.2)	3.1 (2.8−3.4)	2.7 (2.4−3.0)	2.9 (2.7−3.1)
Self‐assessment	16	3.7 (3.4−4.0)	3.5 (3.1−3.8)	3.1 (2.8−3.4)	3.1 (2.9−3.4)

Abbreviations: CI, confidence interval; DDP, disconfirming dimensions percentage.

* *p* < .05

*** *p *< .001.

**Table 2:** Paired *t *test comparing self‐assessment measures (Bias, DDP) in the first encounter and the last (4th) encounter

Paired *t *test	*n*	MD (95% CI)	Effect size (Cohen's *d*)
Bias1−Bias4	16	0.55* (1.0−0.09)	0.64
DDP1−DDP4	16	12.85 (−2.65 to 28.35)	0.77
Faculty assessment1−Faculty assessment4	16	−0.04 (−0.4 to 0.3)	−0.06
Self‐assessment1−self‐assessment4	16	0.51* (0.1−0.9)	0.71

Abbreviations: CI, confidence interval; DDP, disconfirming dimensions percentage; MD, mean difference.

* *p *< .05.

The number of agreed upon strength points of students with faculty in the sessions increased consistently and were 7, 15, 16, and 24, respectively. The difference between the first and the fourth encounter was statistically significant (*p* = .002) according to the Wilcoxon signed‐rank test. The numbers of agreed upon areas of improvements in the sessions were 13, 16, 13, and 10, respectively.

Faculty assessment remained relatively stable across the four encounters, with no significant difference. Self‐assessment, in contrast, decreased consistently in each encounter, with a significant difference between the first and the fourth encounter with a relatively large effect size (*d* = 0.71).

**Figure 3:**
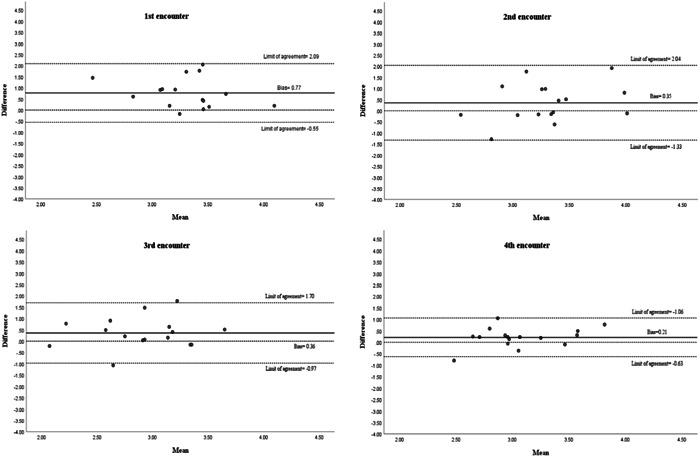
Bland–Altman plots of the differences between self‐assessment and faculty assessment in each encounter against the mean of the two measurements. The bias and limits of agreement are shown as parallel lines to the *X*‐axis

The Bland–Altman graph plots (Figure 3) represent every difference (Bias) between self‐assessment and faculty assessment against the mean of the two measurements in each encounter. The data are relatively symmetrical to the bias line in each encounter plot. The bias scores in the second and the third encounter are almost equal; however, the limits of agreement are narrower in the third encounter by 0.7, and in the fourth encounter, the limits of agreement are narrower than the third encounter by about 1 degree.

**Figure 4:**
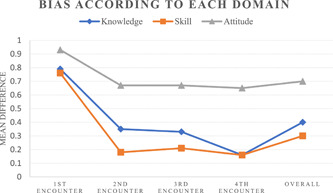
Line graph showing the mean score of the difference (bias) between self‐assessment and faculty assessment in each domain (knowledge, skill, attitude) for each encounter as well as the accumulated score over the 4 encounters

The bias in skills and knowledge assessment consistently decreased after each encounter (Figure 4); they decreased from 0.76 and 0.79, respectively, to 0.16 for both. However, bias in attitude assessment decreased slightly from 0.93 to 0.67 in the second encounter and then reached a plateau at this level. In terms of the overall bias across all encounters, the difference between knowledge and attitude (*n* = 64, mean difference =  0.24, *p* = .013) as well as the difference between overall bias in skills and attitude (*n* = 16, mean difference = 0.34, *p* = .001) were significant; however, the difference between overall bias in knowledge and skills was not significant (*n* = 64, mean difference = 0.10, *p* = .47).

### 3.2 Students' perception of self‐assessment

In each encounter, feedback utility was assessed on the DOPS form on a 5‐point scale. The mean score of all encounters for all students was 4.1 (*n* = 53, SD = 0.76), with a completion rate of 83% for this specific item.

Fourteen out of 16 students completed the postcourse questionnaire. In the postcourse survey, all students agreed that their general experience with self‐assessment was positive and also agreed on its positive impact on their learning. 92.9% (*n* = 13, one student was neutral) agreed that it increased their motivation levels, and 100% agreed that self‐assessment helped them recognize their areas of improvement and strengths better. All students recommended the implementation of this assessment method as part of their clinical training.

Students were asked about the educational impact of self‐assessment in open‐ended questions in the postcourse questionnaire. Seven students pointed out that assessing themselves had a positive impact on their *goal setting*, expressing an orientation toward addressing certain performance aspects. A certain response was echoed by many students:

Becoming self‐satisfied if I did well and this encouraged me to develop my skills more and perform my best, and I had a motive to develop more, learn new things from faculty and avoid making mistakes, which I become firmly aware of thanks to the assessment method

The previous response also contains other themes such as *self‐satisfaction*, *motivation* “encouraged me to,” and *self‐evaluation* “mistakes which I become firmly aware of.” Five students wrote about how the assessment method improved their *self‐evaluation* and showed an understanding of performance *causal attributions*. One student wrote “I recognized my areas of improvement which were posture, patient‐chair positioning and the way I dealt with patients.” Another student wrote about how becoming aware of areas of improvement increased motivation levels: “I learned to admit my areas of improvement with complete honesty without feeling ashamed of them, and this created a great desire to improve more.”

Most respondents asserted that their *attention focusing* improved after using self‐assessment: “I learned to focus more on each step I do during the procedure,” “I started paying attention to steps I did not take into account before,” “I learned to pay attention to all aspects mentioned in the assessment form not just the final shape of the restoration.” Two students wrote that they started noticing certain steps that they previously neglected during the procedure, and this refers to improved *self‐recording* ability.

Students also reported that higher levels of motivation were associated with an increased focus on improving clinical performance (goal setting). Furthermore, three students mentioned that noticing *improvement in performance* (*self‐satisfaction*) helped motivate them: “the increased motivation was due to noticing improvement in certain points, and this was achieved without much effort by focusing attention.” Another recurrent theme was the *educational attention* that students received as well as their ability to identify areas of improvement (*self‐judgment*): “having very cooperative faculty who provided a lot of insight and helped us with complete honesty. Further, the idea of self‐assessment itself makes one aware of his/her own areas of improvement and strengths, and at the same time increases the drive to address all areas that need improvement as much as possible.” Four respondents expressed an increasing *intrinsic interest* in practicing restorative dentistry.

Regarding the challenges of self‐assessment, most students referred to the limited time and pressure due to the high number of students and lack of dental equipment in the department. One student talked about the very high standards in the grading rubric that caused frustration due to the inability to meet these standards, considering the difficult logistic conditions and circumstances at the Faculty of Dentistry in Damascus University. Students also assessed the pressure that the assessment method added to their clinical training on a 5‐point scale (1 = *no pressure*, 5 = *extreme pressure*), and the mean score was 2.64 (*n* = 14, SD = 1.0).

## 4 DISCUSSION

The null hypothesis of the current study was rejected, as there was a significant decrease in the gap between self‐assessment and faculty assessment between the first and the last encounter. Students also became more able to identify strengths areas; however, there was no significant improvement in their ability to exactly pinpoint areas of improvement. Students' perception of self‐assessment was very positive and indicated awareness of improvement in self‐assessment, as well as improved attention focusing, self‐recording, goal‐setting skills, and motivation. An unexpected finding that did not relate to the study hypothesis was that students' performance remained relatively stable across the four encounters despite the improvement in students' self‐assessment.

The narrower limits of agreement in the fourth encounter in Figure 3 indicate lower variability and a smaller margin; this shows higher agreement between students' self‐assessment and faculty assessment in the fourth encounter in comparison with the previous encounters. This improvement in self‐assessment stands in contrast to earlier findings (Curtis et al., 2008; Satheesh et al., 2015). It has been previously suggested that students' self‐assessment ability is relatively stable (T. Fitzgerald, 1997; J. T. Fitzgerald et al., 2003). However, the findings of the current study suggest otherwise. A possible explanation for this is that the students in the previous studies were not formally trained on how to perform self‐assessment. Some authors (Knight et al., 1990; Tuncer et al., 2015) argued that focused systematic training of students in self‐assessment has the potential to lead to improvements in self‐assessment. This argument is consistent with the results obtained in the current study. The students' relatively stable performance levels could indicate that students require more focused practice to achieve better scores, especially in communication skills and professionalism, where students' scores were substandard. Students' poor ability to pinpoint areas of improvement in comparison with strengths suggests that further assistance might be needed so that students become more able to identify the exact areas compromising their performance evaluation.

**Figure 5:**
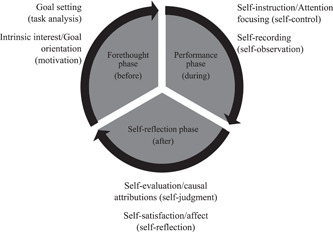
The three phases of self‐regulated learning according to Zimmerman's model. Processes (in brackets) and subprocesses, which were discovered during the thematic analysis in this study, are listed next to their respective phase

Students' very positive perception of self‐assessment further supports the argument that students value practicing self‐assessment and this activity is worthwhile if presented appropriately (Jackson & Murff, 2011). It can also be noticed that students' responses were mainly referring to an improvement in SRL processes or subprocesses as described by Zimmerman (2000) (Artino & Jones, 2013). Most themes were related to one of the three phases of SRL (Figure 5). It can be argued from the conducted qualitative analysis that improvement in self‐assessment ability also affected other processes of SRL according to students' perception; this corroborates the idea that the three phases of SRL are interlocked and mutually dependent. Self‐reflection processes will affect forethought processes, which in turn will affect performance processes. In the performance phase, students become more able to self‐record themselves, and this impacts the self‐reflection processes and so forth (Cleary et al., 2012; Zimmerman, 2000). Previous studies investigating writing revision skills found a high correlation between students' use of different self‐regulatory skills and self‐reflection (Zimmerman & Kitsantas, 1999).

The limitations of this study were the small sample size, the convenience sampling, the lack of a control group, and the lack of inter‐ and intrarater reliability statistics as they were not quantified before the study. However, faculty received extensive training in conducting assessment, and the grading rubric served as the main guide. There are also a number of uncontrolled variables familiar to the clinical settings that could have affected our results. For instance, concordance between faculty and students' assessment could have affected the results; however, we attempted to minimize concordance between students and a single faculty assessor by rotating faculty among students and having students assessed by different assessors in different encout. It cannot be stated conclusively that training students to self‐assess was responsible for reducing the gap between self‐assessment and faculty assessment. Future research should address these limitations directly in order to confirm the validity and reliability of the findings of this study. The contribution of this study lies in the fact that it addressed many limitations that previous studies had such as the lack of formal self‐assessment training and assessor calibration, being limited to a single encounter, and not using or reporting the use of a structured assessment form accompanied by a detailed grading rubric (Mays & Branch‐Mays, 2016). Another strength of this study was providing information on student dentists' perception of self‐assessment; the majority of research in dental education has not treated students' perception of self‐assessment in much detail (Mays & Branch‐Mays, 2016).

## 5 CONCLUSION

This study has provided some evidence to support the hypothesis that self‐assessment of dental students could be bridged with faculty assessment through systematic and deliberate training. Future longitudinal research using a larger sample size is still required to further explore whether self‐assessment can be actively bridged with faculty assessment. It could also be of interest to investigate how self‐assessment affects other processes of self‐regulated learning.

## AUTHOR CONTRIBUTIONS

**Ghaith Alfakhry**: Conceptualization (lead); data curation (lead); formal analysis (lead); investigation (lead); methodology (lead); project administration (lead); supervision (lead); visualization (lead); writing—original draft preparation (lead); writing—review and editing (lead). **Khattab Mustafa**: Project administration; resources; supervision; writing—review and editing. **M. Abdulhadi Alagha**: Methodology; visualization; writing—review and editing. **Hussam Milly**: Methodology; resources; writing—review and editing. **Mayssoon Dashash**: Writing—review and editing. **Issam Jamous**: Methodology; supervision; visualization; writing—review and editing.

## CONFLICTS OF INTEREST

The authors declare no conflicts of interest.

## Supporting information

Supporting information

## Data Availability

The data that support the findings of this study are available from the corresponding author upon reasonable request.

## References

[cre2567-bib-0001] Abdalla, R. , Bishop, S. S. , Villasante‐Tezanos, A. G. , & Bertoli, E. (2021). Comparison between students' self‐assessment, and visual and digital assessment techniques in dental anatomy wax‐up grading. European Journal of Dental Education, 25(3), 524–535. 33188546 10.1111/eje.12628

[cre2567-bib-0002] Almohaimede, A. A. (2021). Comparison between students' self‐evaluation and faculty members' evaluation in a clinical endodontic course at King Saud University. European Journal of Dental Education ​ ​. https://onlinelibrary.wiley.com/doi/10.1111/eje.12733 10.1111/eje.12733 34870874

[cre2567-bib-0003] Artino, Jr., A. R. , & Jones, K. D. (2013). AM last page: Self‐regulated learning—A dynamic, cyclical perspective. Academic Medicine, 88(7), 1048. 23799447 10.1097/ACM.0b013e3182953763

[cre2567-bib-0004] Bland, J. M. , & Altman, D. G. (1999). Measuring agreement in method comparison studies. Statistical Methods in Medical Research, 8(2), 135–160. 10501650 10.1177/096228029900800204

[cre2567-bib-0005] Chambers, D. W. , & LaBarre, E. E. (2014). The effects of student self‐assessment on learning in removable prosthodontics laboratory. Journal of Dental Education, 78(5), 668–680. 24789827

[cre2567-bib-0006] Cho, G. C. , Chee, W. W. , & Tan, D. T. (2010). Dental students' ability to evaluate themselves in fixed prosthodontics. Journal of Dental Education, 74(11), 1237–1242. 21045229

[cre2567-bib-0007] Cleary, T. J. , Callan, G. L. , & Zimmerman, B. J. (2012). Assessing self‐regulation as a cyclical, context‐specific phenomenon: Overview and analysis of SRL microanalytic protocols. Education Research International, 2012, 1–19.

[cre2567-bib-0008] Cleary, T. J. , Durning, S. , Gruppen, L. , Hemmer, P. , & Artino, A. (2013). Self‐regulated learning in medical education. Oxford Textbook of Medical Education, 1, 465–467.

[cre2567-bib-0009] Cohen, J. (2013). Statistical power analysis for the behavioral sciences. Academic Press.

[cre2567-bib-0010] Commission on Dental Accreditation . (2013). Accreditation standards for dental education programs. American Dental Association.

[cre2567-bib-0011] Cuevas‐Nunez, M. C. , Pulido, M. T. , Harpe, S. , Stein, A. B. , & Lempicki, K. (2022). Assessment of communication and physical exam skills: A comparison of students, faculty and standardized patients. Journal of Dental Education ​ ​. 10.1002/jdd.12892 35181888

[cre2567-bib-0012] Curtis, D. A. , Lind, S. L. , Dellinges, M. , Setia, G. , & Finzen, F. C. (2008). Dental students' self‐assessment of preclinical examinations. Journal of Dental Education, 72(3), 265–277. 18316530

[cre2567-bib-0013] Dilbone, D. , Wynkoop, B. , Delgado, A. , Nascimento, M. , Echeto, L. , & Behar‐Horenstein, L. (2016). Clinical assessment in operative dentistry. MedEdPORTAL, 12 ​. https://www.mededportal.org/doi/10.15766/mep_2374-8265.10369

[cre2567-bib-0014] Epstein, R. M. , Siegel, D. J. , & Silberman, J. (2008). Self‐monitoring in clinical practice: a challenge for medical educators. Journal of Continuing Education in the Health Professions, 28(1), 5–13. 18366128 10.1002/chp.149

[cre2567-bib-0015] Eva, K. W. , & Regehr, G. (2011). Exploring the divergence between self‐assessment and self‐monitoring. Advances in Health Sciences Education, 16(3), 311–329. 21113820 10.1007/s10459-010-9263-2 PMC3139875

[cre2567-bib-0016] Evans, A. W. , Leeson, R. M. , & Petrie, A. (2007). Reliability of peer and self‐assessment scores compared with trainers' scores following third molar surgery. Medical Education, 41(9), 866–872. 17727527 10.1111/j.1365-2923.2007.02819.x

[cre2567-bib-0017] Field, P. , & Morse, J. (1985). Nursing research: The application of qualitative methods. Aspen Systems Corporation.

[cre2567-bib-0018] Fitzgerald, J. T. , White, C. B. , & Gruppen, L. D. (2003). A longitudinal study of self‐assessment accuracy. Medical Education, 37(7), 645–649. 12834423 10.1046/j.1365-2923.2003.01567.x

[cre2567-bib-0019] Fitzgerald, T. (1997). Medical student self‐assessment abilities: Accuracy and calibration. The Annual Meeting of the American Educational Research Association in Chicago, IL, ERIC. https://eric.ed.gov/?id=ED410296

[cre2567-bib-0020] Google . *Google Forms*. https://www.google.com/forms/about/

[cre2567-bib-0022] IBM Corp. (2019). *IBM SPSS statistics for Windows. Version 26.0*.

[cre2567-bib-0023] Jackson, S. C. , & Murff, E. J. T. (2011). Effectively teaching self‐assessment: Preparing the dental hygiene student to provide quality care. Journal of Dental Education, 75(2), 169–179. 21293039

[cre2567-bib-0024] Jonsson, A. , & Svingby, G. (2007). The use of scoring rubrics: Reliability, validity and educational consequences. Educational Research Review, 2(2), 130–144.

[cre2567-bib-0025] Knight, G. W. , Guenzel, P. , & Fitzgerald, M. (1990). Teaching recognition skills to improve products. Journal of Dental Education, 54(12), 739–742. 2246401

[cre2567-bib-0026] Lanning, S. K. , Brickhouse, T. H. , Gunsolley, J. C. , Ranson, S. L. , & Willett, R. M. (2011). Communication skills instruction: An analysis of self, peer‐group, student instructors and faculty assessment. Patient Education and Counseling, 83(2), 145–151. 20638816 10.1016/j.pec.2010.06.024

[cre2567-bib-0027] MAXQDA 2020 . (2019). https://www.maxqda.com/

[cre2567-bib-0028] Mays, K. A. , & Branch‐Mays, G. L. (2016). A systematic review of the use of self‐assessment in preclinical and clinical dental education. Journal of Dental Education, 80(8), 902–913. 27480701

[cre2567-bib-0029] Mays, K. A. , & Levine, E. (2014). Dental students' self‐assessment of operative preparations using CAD/CAM: A preliminary analysis. Journal of Dental Education, 78(12), 1673–1680. 25480283

[cre2567-bib-0030] Microsoft Excel . (2016). https://www.microsoft.com/en-us/microsoft-365/excel

[cre2567-bib-0031] Nance, E. T. , Lanning, S. K. , & Gunsolley, J. C. (2009). Dental anatomy carving computer‐assisted instruction program: An assessment of student performance and perceptions. Journal of Dental Education, 73(8), 972–979. 19648568

[cre2567-bib-0032] Patel, S. A. , Halpin, R. M. , Keosayian, D. L. , Streckfus, C. F. , Barros, J. A. , Franklin, D. R. , Quock, R. L. , Jeter, C. B. , & Franklin, A. (2020). Impact of simulated patients on students' self‐assessment of competency in practice of geriatric dentistry. Journal of Dental Education, 84(8), 908–916. 32394449 10.1002/jdd.12176

[cre2567-bib-0033] Saadé, J. M. , El‐Khatib, W. , Chedid, N. R. , Makzoumé, J. E. , El‐Halabi, M. T. , & El‐Hage, F. (2021). Effect of self‐assessment in a removable prosthodontics preclinical course on skills and competence. Journal of Dental Education ​ ​. 10.1002/jdd.12821 34761392

[cre2567-bib-0034] Satheesh, K. M. , Brockmann, L. B. , Liu, Y. , & Gadbury‐Amyot, C. C. (2015). Use of an analytical grading rubric for self‐assessment: A pilot study for a periodontal oral competency examination in predoctoral dental education. Journal of Dental Education, 79(12), 1429–1436. 26632297

[cre2567-bib-0035] Tricio, J. , Woolford, M. , Thomas, M. , Lewis‐Greene, H. , Georghiou, L. , Andiappan, M. , & Escudier, M. (2015). Dental students' peer assessment: A prospective pilot study. European Journal of Dental Education, 19(3), 140–148. 25168409 10.1111/eje.12114

[cre2567-bib-0036] Tuncer, D. , Arhun, N. , Yamanel, K. , Çelik, Ç. , & Dayangaç, B. (2015). Dental students' ability to assess their performance in a preclinical restorative course: Comparison of students' and faculty members' assessments. Journal of Dental Education, 79(6), 658–664. 26034030

[cre2567-bib-0037] Udovičić, M. , Baždarić, K. , Bilić‐Zulle, L. , & Petrovečki, M. (2007). What we need to know when calculating the coefficient of correlation? Biochemia Medica, 17(1), 10–15.

[cre2567-bib-0038] Wieck, M. M. , McLaughlin, C. , Chang, T. P. , Rake, A. , Park, C. , Lane, C. , Burke, R. V. , Young, L. C. , Cleek, E. A. , Morton, I. , Goodhue, C. J. , Burd, R. S. , Ford, H. R. , Upperman, J. S. , & Jensen, A. R. (2018). Self‐assessment of team performance using T‐NOTECHS in simulated pediatric trauma resuscitation is not consistent with expert assessment. The American Journal of Surgery, 216(3), 630–635. 29366483 10.1016/j.amjsurg.2018.01.010 PMC7169991

[cre2567-bib-0039] Zimmerman, B. J. (2000). Attaining self‐regulation: A social cognitive perspective. In M. Boekaerts , M. Zeidner , & P. R. Pintrich (Eds.), Handbook of self‐regulation (pp. 13–39). Elsevier.

[cre2567-bib-0040] Zimmerman, B. J. , & Kitsantas, A. (1999). Acquiring writing revision skill: Shifting from process to outcome self‐regulatory goals. Journal of Educational Psychology, 91(2), 241–250.

