# Supplementary material for: Bridging the gap between self‐assessment and faculty assessment of clinical performance in restorative dentistry: A prospective pilot study
Source: Clin Exp Dent Res. 2022 Apr 6;8(4):883–92. doi: 10.1002/cre2.567 (PMC9382034; doi:10.1002/cre2.567)
Supplement: Supplementary file 1 — Supporting information [file CRE2-8-883-s001.docx]

**Supplementary material**

**Table 3.** Paired t-test showing the mean difference (bias) in each dimension of each domain (Knowledge, Skill, Attitude) across the four encounters. MD: mean difference. CI: confidence interval. **P*<0.05, ***P*<0.01, ****P*<0.001

|  | 1^st^ session | 2^nd^ session | 3^rd^ session | 4^th^ session |
| --- | --- | --- | --- | --- |
|  | MD (95% CI) | MD (95% CI) | MD (95% CI) | MD (95% CI) |
| Knowledge |  |  |  |  |
| 1. Clinical assessment, diagnosis and treatment plan | 1.1***(0.5 to 1.6) | 0.2(-0.4 to 0.7) | 0 (0.5 to -0.5) | -0.4(-0.1 to 1) |
| 2. Demonstrates understanding of indications, dental materials and used technique | 0.9***(0.6 to 1.2) | -0.06(-0.5 to 0.4) | -0.07(-0.6 to 0.5) | -0.06(-0.4 to 0.3) |
| 10. Seeking help when appropriate. | 0.3(-0.2 to 0.8) | 0.5(-0.5 to 1.3) | 0.5(-0.22 to 1.1) | -0.1(-0.6 to 0.3) |
| Skills |  |  |  |  |
| 6. Chair, patient and dentist’s position | 0.5(-0.2 to 1.3) | -0.06(-0.6 to 0.5) | -0.1(-0.8 to 0.5) | -0.06(-0.6 to 0.5) |
| 7. Preparation for the restoration | 1.1**(0.5 to 1.8) | 0.1(-0.3 to 0.6) | 0.1(-0.3 to 0.5) | 0.4(-0.07 to 0.9) |
| 8. Restoration | 0.5*(0.04 to 1) | -0.005(-0.6 to 0.4) | 0.5(-0.04 to 1.1) | 0.1(-0.22 to 0.5) |
| Attitude |  |  |  |  |
| 3. Obtaining patient consent after explaining the procedure and possible complications | 1.2*(0.3 to 2.1) | 0.3(-0.1 to 1.1) | 0.7*( 0.05 to 1.3) | 0.5*(0.02 to 1.0) |
| 4. Pre-procedural preparation | 1.7***( 0.9 to 2.5) | 1.3*( 0.05 to 2.3) | 1.2*(0.3 to 2.2) | 1.5**(0.5 to 2.4) |
| 5. Pain, anxiety management and communication skills | 0.9*( 0.2 to 1.6) | 0.3(-0.2 to 1.3) | 0.7*(0.05 to 1.3) | 0(-0.6 to 0.6) |
| 9. Infection control and safe disposal of biohazard materials and sharp tools | 0.6(-0.02 to 1.2) | 0.3(-0.4 to 1.1) | 0.5(-0.1 to 1.1) | 0.3(-0.2 to 0.8) |
| 11. Patient education | 1.8***(1.0 to 2.7) | 1.3**( 0.5 to 1.9) | 1.0**( 0.3 to 1.8) | 1.0**(0.3 to 1.6) |
| 12. Organization, efficiency and time management | 0.1(-0.4 to 0.7) | 0(-0.7 to 0.7) | -0.06(-0.6 to 0.5) | 0.06(-0.7 to 0.8) |
| 13. Professionalism | 0.06(-0.4 to 0.5) | 0.1(-0.4 to 0.7) | 0.06(-0.5 to 0.6) | 0.2(-0.1 to 0.5) |
| 14. Overall ability to complete the procedure | 0.4(-0.6 to 1.4) | 0.28(-0.4 to 0.9) | 0.35(-0.2 to 0.9) | -0.1(-0.4 to 0.1) |

**Table 4.** Questionnaire assessing students’ perception of the self-assessment protocol after using the Self-DOPS on four encounters.

| To what extent you agree with the following statements | Strongly disagree | Disagree | Neutral | Agree | Strongly agree |
| --- | --- | --- | --- | --- | --- |
| Your general clinical experience was positive | 0 | 0 | 0 | 21.4% | 78.6% |
| Allowing students to assess themselves had a positive impact on their learning | 0 | 0 | 0 | 64.3% | 35.7% |
| This assessment method increased your motivation to learn | 0 | 0 | 7.1% | 14.3% | 78.6% |
| This clinical assessment method helped you recognize your weaknesses and strengths better | 0 | 0 | 0 | 28.6% | 71.4% |
| I recommend implementing this assessment method as part of the clinical training | 0 | 0 | 0 | 42.9% | 57.1% |

**General changes to the DOPS assessment form**

The following additions were implemented to the form: first, the grading scale was changed to 5-point criterion-referenced scale (Clear fail, Borderline fail, Borderline pass, Clear pass, Excellent). This scale was used in previous studies that assessed clinical performance.^1^ Second, certain dimensions (items) were added, so they are evaluated. *Item 1. Clinical assessment, diagnosis and treatment plan, and item 11. Patient education* were added. The assessment of technical skill assessment was one dimension (item) in the used template; however, it was subdivided into three distinct dimensions (*Chair, Patient and dentist’s position*, *Preparation for the restoration*, *Restoration*) to increase assessment accuracy and clarity. Thirdly, pain management and communication skills were phrased so they become one dimension rather than being assessed separately as communication skills affect pain and anxiety levels of the patient.^2^ These modifications are in accordance to previous published assessment protocols.^3^

**References**

1. Ringsted C, Østergaard D, Ravn L, Pedersen J, Berlac P, Van der Vleuten C. A feasibility study comparing checklists and global rating forms to assess resident performance in clinical skills. *Medical Teacher*. 2003;25(6):654-658.

2. Armfield JM, Heaton L. Management of fear and anxiety in the dental clinic: a review. *Australian dental journal*. 2013;58(4):390-407.

3. Dilbone D, Wynkoop B, Delgado A, Nascimento M, Echeto L, Behar-Horenstein L. Clinical Assessment in Operative Dentistry. *MedEdPORTAL*. 2016;12
